# Object-Based Thermal Image Segmentation for Fault Diagnosis of Reciprocating Compressors

**DOI:** 10.3390/s20123436

**Published:** 2020-06-18

**Authors:** Rongfeng Deng, Yubin Lin, Weijie Tang, Fengshou Gu, Andrew Ball

**Affiliations:** 1 Beijing Institute of Technology, Zhuhai 519088, China; Yubin.Lin@hud.ac.uk (Y.L.); Weijie.Tang@hud.ac.uk (W.T.); 2 Centre for Efficiency and Performance Engineering, University of Huddersfield, Huddersfield, West Yorkshire HD1 3DH, UK; Fengshou.Gu@hud.ac.uk (F.G.); A.Ball@hud.ac.uk (A.B.)

**Keywords:** reciprocating compressors, fault diagnosis, thermal imaging, support vector machines (SVM)

## Abstract

As an essential mechanical device in many industrial applications, reciprocating compressors have a high demand for operating efficiency and availability. Because the temperature of each part of a reciprocating compressor depends considerably on operating conditions, faults in any parts will cause the variation of the temperature distribution, which provides the possibility to distinguish the fault type of reciprocating compressors by differentiating the distribution using infrared thermal imaging. In this paper, three types of common fault are laboratory experimented in an uncontrolled temperature environment. The temperature distribution signals of a reciprocating compressor are captured by a non-contact infrared camera remotely in the form of heat maps during the experimental process. Based on the temperature distribution under baseline condition, temperature fields of six main components were selected via Hue-Saturation-Value (HSV) image as diagnostic features. During the experiment, the average grayscale values of each component were calculated to form 6-dimension vectors to represent the variation of the temperature distribution. A computational efficient multiclass support vector machine (SVM) model is then used for classifying the differences of the distributions, and the classification results demonstrate that the average temperatures of six main components aided by SVM is a promising technique to diagnose the faults of reciprocating compressors under various operating conditions with a classification accuracy of more than 99%.

## 1. Introduction

As an essential mechanical device in many industrial applications, reciprocating compressors have the advantages of high compression efficiency, a simple device system, and mature technology. Therefore, the reciprocating compressors have a wide range of industrial applications. However, the shortcomings such as too many moving parts and complicated structures can lead to various common faults. In order to avoid causing significant economic losses and casualties, it is very important to diagnose the fault of reciprocating compressor in time.

Among sensing technologies such as acoustic emissions, vibration, acoustics and pressure curve synthesis, vibration-based methods are mainstream and actively studied for different applications such as machine tools [1], production lines [2], wind turbines [3] and so on. For monitoring reciprocating compressors, researchers have made a considerable effort to study different sensing methods for more cost-effective and easier implementing techniques. Abaszade et al. [4] determined the compressor valve fault and piston rod wear by collecting pressure information in the compressor cylinder. Verma et al. [5] combined the universal data-mining model to diagnose multiple faults of reciprocating compressors based on the sound signals collected from only one location. Townsend et al [6] expressed the fault information by extracting multiple features in the time domain, frequency domain and time-frequency domain of the vibration signal, and on this basis realized the detection of the valve fault of the reciprocating compressor. Based on vibration and acoustic analysis, Munari et al. [7] detected and identified the precursor signals of the stall and surge of a multi-stage compressor. Ali et al. [8] studied the variation and sensitivity of acoustic emission parameters under different working conditions of the valve, and proved that the acoustic emission parameters can be used to detect the valve failure of reciprocating compressor. Li et al. [9] also used acoustic emission signals to successfully detect the oil pressure state of diaphragm compressor. Sim et al. [10] proposed a method based on acoustic emission signals to quantify the severity of valve problems of reciprocating compressors. Li et al. [11] presented a new method to record a pressure-volume diagram of reciprocating compressor by measuring piston rod strain, and successfully monitored whether the reciprocating compressor valve is leaking or fluttering. Feng et al. [12] proposed a method for calculating the crankshaft tangential acceleration based on an on-rotor MEMS (Micro-Electro-Mechanical System) accelerometer system, which can detect, locate and diagnose the leakage faults of a reciprocating compressor. Haba et al. [13] studied induction motor current signatures to detect and diagnose faults of reciprocating compressor, which creates a varying load to the motor. These methods are usually based on advanced signal-processing techniques and need to collect multiple parameters, which increases the difficulty of practical applications. Meanwhile, the mapping relationship between these extracted parameters and fault types under different operating condition is complex, and is difficult to interpret. 

Compared with the model-based methods, big data and machine-learning technologies have unique advantages in establishing the relationship between parameters and fault types, and provide new technical ideas for diagnosing the failure of reciprocating compressors. Qi et al. [14] proposed a method based on vibration signal preprocessing and support vector machine (SVM), which achieved better results than the model-based methods in the potential fault diagnosis of compressors. Pichler et al. [15] extracted the features of vibration signals from the difference of a time-frequency spectrogram between the normal and broken valves of different geometries and materials at various compressor loads, and performed fault diagnosis of reciprocating compressor through logistic regression and SVM methods. Zhang et al. [16] combined a novel convolutional deep belief networks and multi-source information to improve the performance of fault diagnosis of reciprocating compressors. Cabrera et al. [17] used a time series of vibration signals collected from the compressor to train a set of long short-term memory (LSTM) models, which is suitable for diagnosing the valve failure of reciprocating compressors. Other fault-diagnosis techniques such as the k-nearest neighbors (KNN) for temperature data [18], artificial neural networks (ANN) or a genetic algorithm for vibration data [19,20] and hybrid deep belief network (HDBN) for pressure, motor current and vibration data [21], ref. [22] have been demonstrated by other researchers. These methods all require a large number of learning samples, and the acquisition of samples, especially the acquisition of unknown faults, is the main dilemma of such methods.

Reciprocating compressors produce a certain amount of heat during the process of compressing gas. Experiments have found that the temperature distribution changes during the early stage of failures in reciprocating engines [23], i.e., the piston ring failure will cause the temperature distribution of the suction and exhaust regions to change [14]. In addition, the failure of the cooling system can cause the local temperature of the compressor to rise rapidly. Similarly, motor failure often leads to an increase in the temperature of the motor body. The characteristics of this temperature distribution variation are closely related to the types of faults [24], which provides opportunities for effective and efficient fault diagnosis of various faults using the temperature measurements i.e., infrared thermal images.

Infrared thermal cameras are a non-contact temperature measuring device that can accurately obtain the surface temperature distribution information of the measured object in real time. According to this function, thermal imaging technology has been widely used in scientific research and industrial production [25,26,27,28,29]. Consequently, this paper proposed a method to validate the feasibility and robustness of using an infrared imaging camera to diagnose the faults of reciprocating compressors based on the intelligent classification method of SVM.

## 2. Experimental Facilities

### 2.1. Test Rig

**Figure 1:**
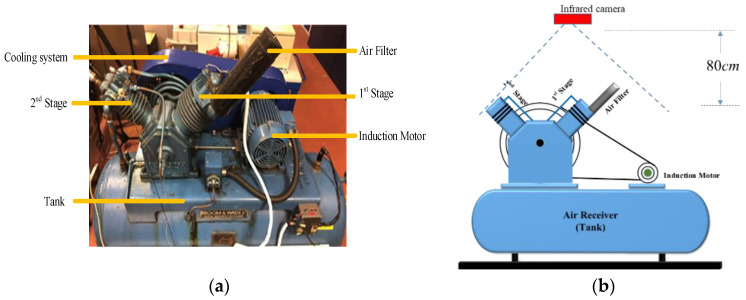
Experiment facilities: (**a**) test rig; (**b**) the positional relationship between the infrared camera and the reciprocating compressor.

In order to verify the feasibility of using the infrared images to identify the faults of reciprocating compressors, experiments were conducted based on a two-stage V-shape reciprocating compressor which is driven by a three-phase, 2.5 kW induction motor through a transmission belt. The structure of the experimental test rig is shown in Figure 1a. A cost-effective infrared camera, FLIR One Pro with a temperature resolution of 0.1 ℃ positioned at 80 cm above the top of the are filter, is used to capture the temperature distribution of the entire system. The positional relationship between the infrared camera and the reciprocating compressor is shown in Figure 1b.

The experiments were conducted under different ambient temperature. Additionally, the camera was located at a constant distance to the compressor when it was running. However, the camera position changed slightly due to vibrations caused by the compressor and human disturbance during installation resulting in a slight image shift during the operation of compressors with different faults. The pressure in the tank rose from atmospheric pressure to about 0.8 MPa (or 120 psi) which is recommended by the compressor manufacturer during the operation. This process lasted for about 10 min with the thermal video recorded.

### 2.2. Fault Simulation

Faults in different parts of reciprocating compressors often induce changes in the temperature field. For example, motor faults will cause changes in the motor temperature distribution. This feature has been widely used in motor fault diagnosis [16,17], according to the gas thermodynamic equation:

PV = vRT(1)

where P represents gas pressure; V represents gas volume; v represents gas quality; T represents gas temperature; R is constant. The speed at which the compressor draws in gas will affect n, while changes in the compressor’s internal pressure will affect P, which will cause changes in the internal gas temperature. Under the effect of heat convection, the temperature distribution of different parts of the compressor will also change accordingly.

**Figure 2:**
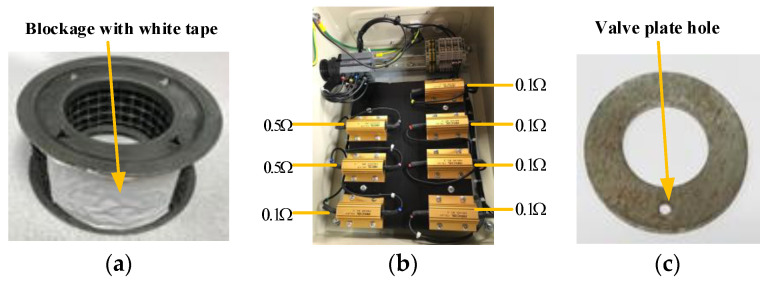
Fault simulation: (**a**) air filter blockage (AFB), (**b**) asymmetrical stator winding (ASW), (**c**) discharge valve leakage (DVL).

According to the above simple analysis, this study experimented in the laboratory with three common faults of reciprocating compressors: air filter blockage (AFB), asymmetrical stator winding (ASW), and discharge valve leakage (DVL). The first fault shown in Figure 2a was set up by simulating an artificial blockage (covering 25% of the air filter with a white tape). The second type of fault was simulated by adding a phase winding resistance using the external resistor bank ($R_{fs}=5\times 0.1\Omega +0.5\Omega =1.0\Omega$), making the stator winding asymmetrical, which is shown in Figure 2b. The third type of fault in Figure 2c was set up by drilling a 2 mm hole in the valve plate, so that the gas can leak into the tank from the second stage in advance, simulating valve leakage into the compressor tank. The number of images recorded in the baseline (BL, represents the state of the reciprocating compressor during normal operation) and the three failure modes during the experiments, and the ambient temperature corresponding to the different cases, are shown in Table 1. Since the FLIR One Pro infrared camera cannot fix the frame rate, the frame rate of the video collected in different experiments cannot be consistent.

## 3. Analysis of Temperature Change of Reciprocating Compressor

### 3.1. Temperature Change Characteristics of Reciprocating Compressor Based on Pseudo-Color Analysis

The higher the object temperature is, the faster the particles move. Consequently, more energy radiates outward at higher temperature and vice versa. Based on this principle, infrared cameras convert the infrared radiation energy of objects into electrical signals, and display them in the form of images. Because color images can express a wider color range than grayscale images, and because the color range that a color image can express is wider than the color range that a grayscale image can express, an infrared image is expressed by converting electrical signals into a color image through a specific process such as rainbow encoding or hot-metal encoding. All of these encoding methods have a one-to-one correspondence between temperature and color, so the temperature of the object can be analyzed by analyzing the color of the image.

**Figure 3:**
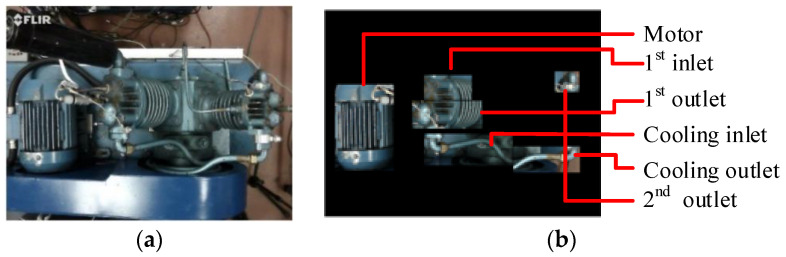
The main regions of images of interest: (**a**) image of the test rig, (**b**) six selected regions.

In order to understand the law of temperature rise in different parts of the reciprocating compressor, six interest regions were selected from color image, which corresponds to the temperatures of the motor (Motor), first stage inlet (1st inlet), first stage outlet (1st outlet), cooling system inlet (Cooling inlet), cooling system outlet (Cooling outlet), second-stage outlet (2nd outlet) respectively, as shown in Figure 3. Moreover, these parts are the core components of the reciprocating compressor. The variation of the temperature in these six regions is highly correlated with the performance of the compressors and any fault that occurred can result in the abnormal variation of the temperature.

**Figure 4:**
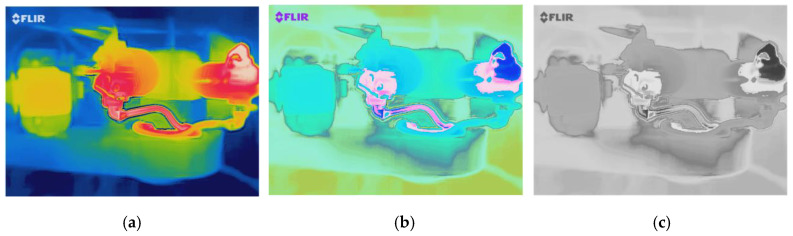
Convert color mode of infrared image: (**a**) infrared image in red, green and blue (RGB) mode, (**b**) infrared image in HSV mode, (**c**) infrared image in Grayscale mode.

Due to the inconsistent surface structure of the selected parts, the rate of temperature rise at different points in the same part are also different. This study ignores the heating process at each point, and focuses on the variation trend of temperature compared with the final state of the temperature at each point. The last frames of the infrared videos obtained under different working conditions was converted to HSV color mode which is shown in Figure 4b, which shows the temperature distribution at balance conditions. It can be seen that edge profiles for different parts in HSV mode are more distinctive. Therefore, the regions for interested objects can be more accurately determined based on HSV images.

**Figure 5:**
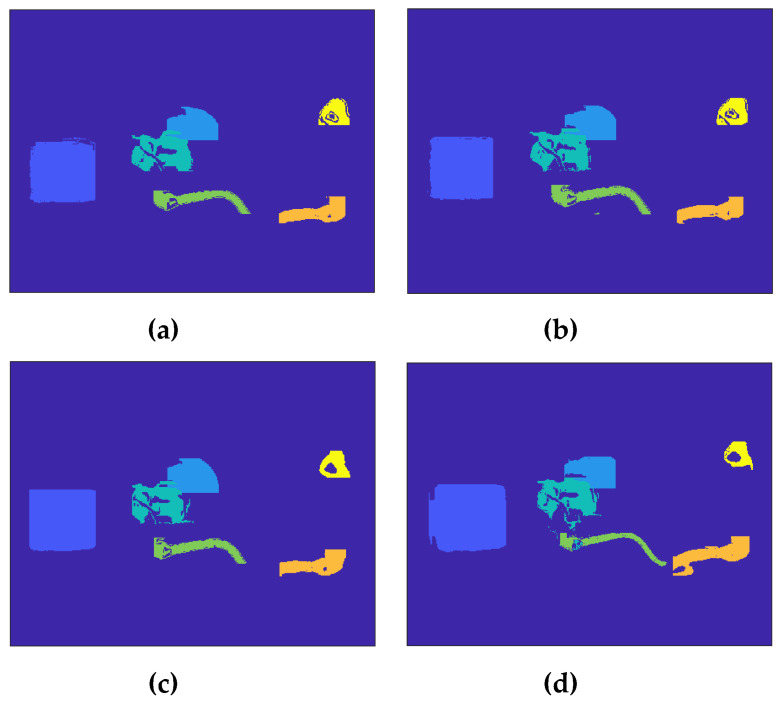
Screening results of points with similar heating rates in six regions under 4 different working conditions: (**a**) BL, (**b**) AFB, (**c**) ASW, (**d**) DVL.

Divide each region according to the threshold in Table 2, the points with similar heating rates in six regions of interest were screened. The results under 4 different working conditions are shown in Figure 5.

**Figure 6:**
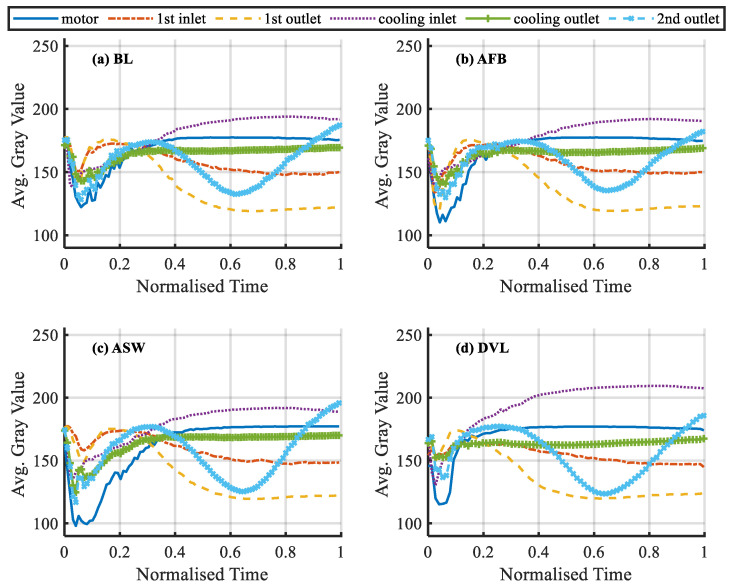
The average grayscale value of the six regions under different working conditions in which the variation process is normalized by operating time (**a**–**d**).

**Figure 7:**
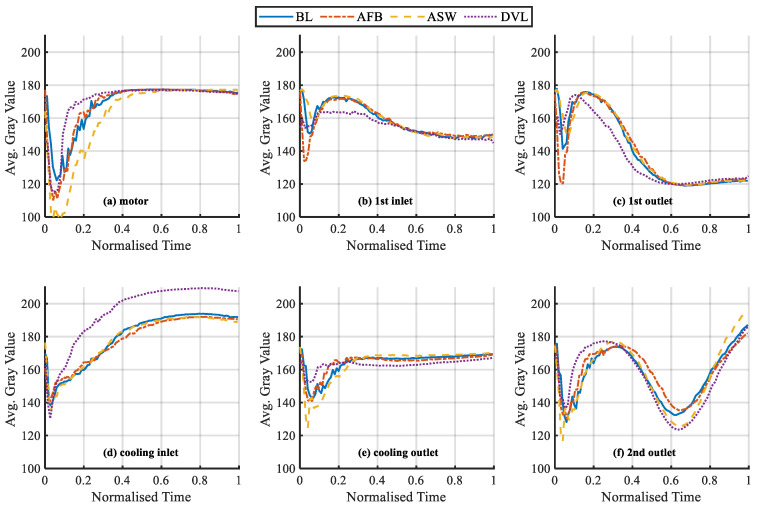
Comparison of the average grayscale values between six regions under different operating conditions (**a**–**f**).

Each frame in the acquired infrared video is converted into a grayscale image as show in Figure 4c. Then the average grayscale value of all points in each region is calculated. Under different working conditions, the variation process of the average grayscale values of six regions after time normalization are shown in the Figure 6 and Figure 7. It can be seen that in the lower operating temperature phases when the normalized time is lower than 0.6, these six variables show significant differences between fault cases. This means that these cases can be separated easily. Meanwhile, in the higher operating temperature range when the time is above 0.6, the differences between variables are not very significant but still show clear differences. Based on these differences it is possible to separate different cases for the entire operating phase. However, because of the complicated change patterns between these six variables during the operating phases, it needs a more advanced method such as SVM for the classification task.

### 3.2. Fault Type Classification Using Support Vector Machine (SVM)

**Figure 8:**
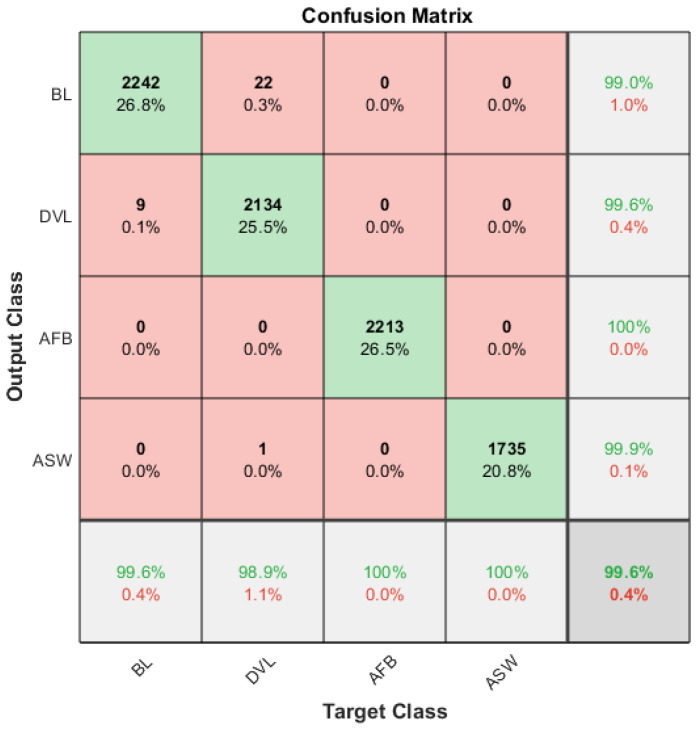
Confusion matrix of the training accuracy.

Although there are clear differences between the grayscale values for the entire operating process, it is not easy to use a simple method to differentiate the four cases. This study chooses a popular SVM algorithm for classifying these complicated data patterns because of its better generalization and nonlinear classification abilities, showing outstanding performances in diagnosing different faults [30]. In particular, a data matrix of 9284 × 6 was constructed from the 9284 images each of which has 6 grayscale values. Then 90% of the datasets with random entrances was selected to train a multiclass SVM model using the classification learner application of Matlab. Choose the refined Gaussian kernel as the kernel function, and the kernel scale is set to 0.61, the box constraint level is set to 1. Moreover, the training data is divided into 10 parts for cross-validation to ensure the generalization ability. The confusion matrix presented in Figure 8 shows that the SVM classifier can reach a correct classification rate as high as 99.6% for the training sets. This indicates that the model trained is acceptable. Moreover, it also shows that datasets extracted include the information for differentiating the complicated temperature distributions.

**Figure 9:**
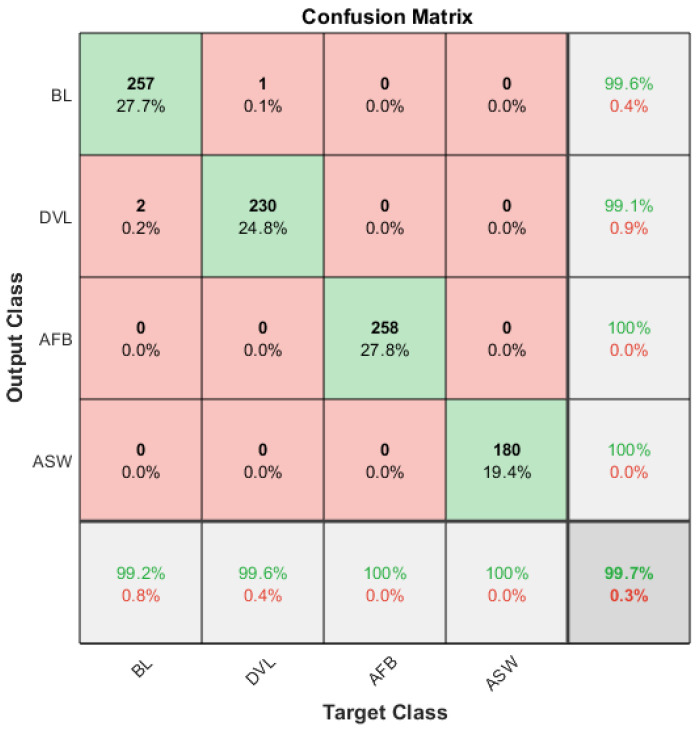
Confusion matrix of the testing accuracy.

Using the trained classifier to classify the test data, the results show that the classification accuracy reaches 99.7%. The testing result is shown in Figure 9 with a confusion matrix. These results furthermore confirm that the proposed method is effective and efficient to detect and diagnose compressor faults in a wide range of operating conditions. 

**Figure 10:**
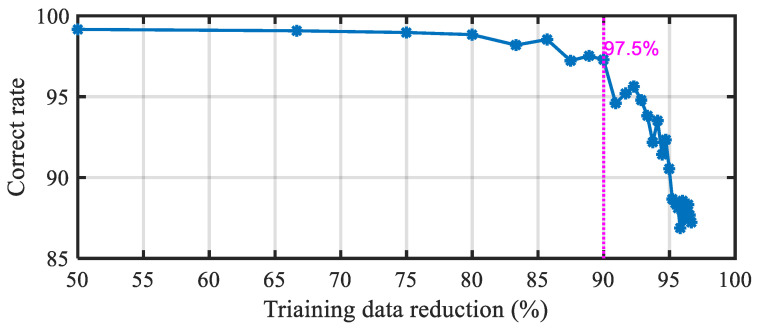
Effects of different proportions of training data on classification accuracy.

In order to further confirm the robustness of the method proposed in this paper, 50% of the data are randomly selected as test data, and the data involved in training are continuously reduced from the remaining data. We use the test data to verify the trained classifiers, and the trend of the proportion of correct classification is shown in Figure 10. As can be seen that using 10% to 50% of the data for training, the correct classification rate remains above 97.5%, even if less than 5% of the data is used for training, correct classification rate is still more than 86%. It can be concluded from this that the method proposed in this paper is highly robust and does not require an infrared camera with a high frame rate.

One of the important merits using the proposed method for the thermal based monitoring is that it needs much less computational effort, compared with the deep learning-based methods [31]. Moreover, this thermal image analysis-based monitoring approach can have more comprehensive monitoring information at a lower cost of instrumentation and processing effort, compared with vibration [3,4,7], acoustics [4,5] and motor current based [8] monitoring methods. Furthermore, the developed supervised learning model in the laboratory environment can be transferred easily into the industrial applications because the variation trend of temperature is usually less influenced by the environments and only relies on the working principle of the reciprocating compressors if compared with other dynamic measurements including vibration, acoustics, and instantaneous current signals. 

According to basic common sense, the temperature range (typically from 50 °C to 85 °C) of the compressor during normal operation is usually distinctive and relatively stable. In this study, the SVM classifier was trained using the all dataset from the entire process, and it allows the faults to be detected from a cold start process. However, when the compressor has a warm-up cycle, the detection performance probably will low for a short period before the temperature becomes stabilized. Once the temperature becomes stable, the distribution of temperature will follow the profiles and thus the detection performance will be recovered.

Of course, we also note that the ambient temperature will have a significant impact on our method. The actual working environment of the compressor has a larger temperature change than that under-laboratory conditions. To maintain the performance of fault detection, the SVM classier should be trained with such wide ambient temperatures. However, it needs a further study to evaluate the robustness to ambient temperature ranges. According to the current study in which the SVM can perform sufficiently well for the ambient temperature from 20.5–24.7 °C, it may need only a few ambient temperature points to have a robust SVM trained, which is not only feasible but also efficient as the object-based thermal image segmentation, proposed in this study needs little computation effort. 

## 4. Conclusions

This paper proposed a non-intrusive diagnosis method based on thermal images and SVM classifiers for the condition monitoring of reciprocating compressors. Six image regions were selected based on the key subsystems of the reciprocating compressor. The average grayscale values of each region in the grayscale infrared images was calculated to form 6-dimensional feature vectors, which highlights the differences of the temperature variation between subsystems while significantly reducing the data dimensions. An easy implementation classifier, SVM, was employed for fault type classification under different operating conditions. The statistical average accuracy of the classification can reach up to 99.7%, which verifies this method has high accuracy and reliability. The varying thermal information of the core components in compressors is highly correlated to the working principle. Therefore, the proposed intelligent method is potentially transferred into the industrial applications. To summarize, the infrared camera, as a non-intrusive and non-contact measuring device, incorporating with engineering knowledge-based data processing, has promising applications for the intelligent condition monitoring and fault diagnosis of reciprocating compressors.

## Figures and Tables

**Table 1:** The number of images recorded in various failure modes and the ambient temperature.

Fault Modes	Frame Rate	Duration	Number of Images	Ambient Temperature (°C)
BL	4.04 frame/s	10′22″	2515	23.5
AFB	4.09 frame/s	10′04″	2393	23.1
ASW	3.92 frame/s	10′10″	2469	20.5
DVL	3.02 frame/s	10′31″	1907	24.7

**Table 2:** The threshold for segmentation of each region.

Region	Hue Range	Saturation Range	Value Range
moto	——	——	>0.8
1st inlet	<0.1	——	——
1st outlet	>0.95	——	——
Cooling inlet	<0.1	——	——
Cooling outlet	<0.2	——	>0.7
2nd outlet	<0.09	<0.6	——
